# Another Medical College in Texas

**Published:** 1897-08

**Authors:** 


### EDITORIAL DEPARTMENT.

F. E. DANIEL, M. D., Editor.
S. E. HUDSON, M. D., Managing Editor.
A. J. SMITH. M. D., Galveston, Associate Editor.

Published Monthly at Austin, Texas, by Drs. Daniel and Hudson. Subscription price $2.00 a year in advance.

Eastern Agents: The Monihan-Fairchild Special Agency, St. Paul Building, New York City.

Official organ of the West Texas Medical Association, the Houston District Medical Association, the Austin District Medical Society, Brazos Valley Medical Association, the Galveston County Medical Society, and several others.

### ANOTHER MEDICAL COLLEGE IN TEXAS.

There is no limit to the patriotism and enterprise of Texas doctors. No Texas town is now thought to be complete without its medical college, and the physicians of Weatherford keenly alive to this fact, have hastened to supply a long felt want. They have introduced however an innovation, which will surely be appreciated by the farmer class—whose sons will be made doctors: towit: they will take pay "in kind." We give below the announcement of the first session of the COLLEGE OF PHYSICIANS AND SURGEONS OF WEATHERFORD.

As medical colleges seem to be the fad in every village in the State, we beg leave to submit a prospectus.

PROSPECTIVE.

The first annual opening of the College of Physicians and Surgeons, of Weatherford, Texas, will take place the first Monday in September, 1897.

Introductory lecture will be delivered by Prof. Oliver Morse, M. D., which will be largely devoted to the religious and moral duties of the medical student.

Being aware of the long standing necessity of a more extended, accessible and thorough medical education for the young men of our country, we, the following medical gentlemen, have organized ourselves into a faculty for the purpose of offering the above and much needed opportunities to the young men of our land.

Owing to the great demand for business property in our city, we find it difficult to secure a suitable building for our first session; therefore, we have secured the upper story of Blackwell's livery stable, corner Spring street and York avenue, as the only place at our command for the present.

The instruction will be both clinical and didactic. Our clinical and hospital facilities are unequalled. The county jail affords all needed hospital instructions.

Our Professor of Medicine, Prof. O. Morse, has the honor of being county physician, therefore, has absolute control of all sickness and infirmities at the county poor farm, where he will hold tri-weekly clinics, at which time the medical class will be carried free of charge to the poor farm, infantry style. In addition to Prof. Morse's other and varied duties, he will endeavor to find the time to deliver once a week a lecture on Moral Philosophy, as pertains to the duties of medical students and physicians.

To enable poor young men to secure medical education, the faculty, not being pressed financially, will accept in lieu of cash, cord wood, Johnson grass, chickens, eggs, or any other commodity the faculty can consume.

To be eligible for matriculation, the candidate must be advanced as far as "Baker" in the old blue-backed speller and know the multiplication table thoroughly. The candidate must be of good moral character, and at least have no present indictment pending, and in event of having served a term in the penitentiary, he must have had his citizenship restored by the governor.

### QUALIFICATIONS FOR GRADUATION.

The candidate must be at least 21 years of age, and must have attended three courses at a regular medical college, the last of which must have been in this institution, and no term to be of less than three weeks duration.

### TEXT-BOOKS.

Gunn's Family Medicine.

Treatise on Pierce's Golden Discovery.

J. Wilford Hall on Constipation.

### FACULTY.

O. Morse, M. D., D. D., Professor of Principles and Practice of Medicine. Dean.

A. R. Barry, M. D., L. L. D., Professor of Medical Jurisprudence and Toxicology.

G. H. Sandefer, M. D., Professor of Principles and Practice of Surgery and Clinical Surgery.

L. Lanier, M. D., Professor of Pathology and Bacteriology.

Alonzo Sims, M. D., Professor of Diseases of Women and Clinical Gynecology.

Alf. Irby, M. D., Professor of Obstetrics.

Wm. Maddox, M. D., Ph. D., Professor of Chemistry.

L. F. Ferry, M. D., Professor of Rectal and Genito-Urinary Surgery.

I. E. Smith, M. D., Professor of Diseases of the Eye, Ear, Nose and Throat.

C. MacNelly, M. D., Professor of Anatomy.

——————, Professor of Materia Medica and Therapeutics.

L. Wilder, M. D., Demonstrator of Anatomy.

B. Brazolton, M. D., Assistant Demonstrator.

Chas. Glaze (colored), janitor.

We have exhausted the profession in the town in the faculty, rnd as soon as a new one strikes town we will fill the vacancy.

[Later: A new man hearing that there was a vacant professorship at Weatherford, hastened to locate there to secure it. He went from the flourishing city of Hogwallow, a town too slow for him; it had no medical college.—Ed.]

